# What utility scores do mental health service users, healthcare professionals and members of the general public attribute to different health states? A co-produced mixed methods online survey

**DOI:** 10.1371/journal.pone.0205223

**Published:** 2018-10-23

**Authors:** Chris Flood, Sally Barlow, Alan Simpson, Amanda Burls, Amy Price, Martin Cartwright, Stefano Brini, Service User and Carer Group Advising on Research (SUGAR) members

**Affiliations:** 1 Centre for Mental Health Research, School of Health Sciences, University of London, London and East London NHS Foundation Trust, London, United Kingdom; 2 Centre for Health Services Research, School of Health Sciences, University of London, London, United Kingdom; 3 Department of Continuing Education, University of Oxford, Oxford, United Kingdom; Yokohama City University, JAPAN

## Abstract

**Background:**

Utility scores are integral to health economics decision-making. Typically, utility scores have not been scored or developed with mental health service users. The aims of this study were to i) collaborate with service users to develop descriptions of five mental health states (psychosis, depression, eating disorder, medication side effects and self-harm); ii) explore feasibility and acceptability of using scenario-based health states in an e-survey; iii) evaluate which utility measures (standard gamble (SG), time trade off (TTO) and rating scale (RS)) are preferred; and iv) determine how different participant groups discriminate between the health scenarios and rank them.

**Design and methods:**

This was a co-produced mixed methods cross-sectional online survey. Utility scores were generated using the SG, TTO and RS methods; difficulty of the completing each method, markers of acceptability and participants’ preference were also assessed.

**Results:**

A total of 119 participants (58%) fully completed the survey. For any given health state, SG consistently generated higher utility scores compared to RS and for some health states higher also than TTO (i.e. SG produces inflated utility scores relative to RS and TTO). Results suggest that different utility measures produce different evaluations of described health states. The TTO was preferred by all participant groups over the SG. The three participant groups scored four (of five) health scenarios comparably. Psychosis scored as the worst health state to live with while medication side-effects were viewed more positively than other scenarios (depression, eating disorders, self-harm) by all participant groups. However, there was a difference in how the depression scenario was scored, with service users giving depression a lower utility score compared to other groups.

**Conclusion:**

Mental health state scenarios used to generate utility scores can be co-produced and are well received by a broad range of participants. Utility valuations using SG, TTO and RS were feasible for use with service users, carers, healthcare professionals and members of the general public. Future studies of utility scores in psychiatry should aim to include mental health service users as both co-investigators and respondents.

## Introduction

Mental ill health is a key contributor to the burden of disease [1] costing an estimated £70-£100 billion per year in the United Kingdom (UK), equivalent to 4.5% of gross domestic product (GDP) [2]. Over half of this cost relates to reduced quality of life [3]. There is a need to prioritise interventions that are cost-effective and target health states that service users report have the greatest impact on their lives.

Traditionally, health economists and policy-makers use health utilities to estimate treatment cost-effectiveness and inform prioritizing decision-making [4]. Utility is a weighted and scaled method of quantifying a person’s preference for an experienced or hypothetical health state [5]. Utility scores are obtained by asking people to evaluate their preference for living in particular health states (e.g., depression) or experiencing a health-related event (e.g., medication side effects). This evaluation may draw on a person’s current or past experience, or their imagining of what it would be like to live with the health state in the future [6]. Service users who live with mental illness/distress and receive mental health treatments are well-placed to inform policymakers on the impact of mental ill health on their quality of life.

Utility scores can be elicited using various methodologies including time trade-off (TTO), standard gamble (SG) and rating scales (RS) [7]. These methods ask people to consider hypothetical health states and either trade an improvement in health for a reduction in time alive (TTO) or a greater risk of death (SG), or to rate the health state on a scale (RS). Several factors are important when choosing a utility method to use. These include their psychometric properties such as: validity (does the method elicit a true preference for the health state?); reliability (does the method elicit reproducible scores?); feasibility (is the method practical for the target population and setting?); and acceptability for the target population. In situations where there is relatively little experience with making health state valuations it is desirable to employ several methods in parallel to determine which is the most suitable [8]. However, when employed in parallel, different methods can yield different utility scores raising legitimate concerns about how patients, commissioners and policy-makers should use the evidence from different utility methods to guide their decisions [9, 10].

The SG is considered the ‘gold standard’ method because it includes an element of uncertainty, thought to reflect real world uncertainty over decisions about health and healthcare. The TTO and RS do not involve uncertainty but rather derive utility values which may be transformed to utility scores [11]. Some studies report that the SG method generates higher scores (indicating more positive evaluations, or less negative evaluations, of the health state) than TTO and RS [12]. Similarly some have found that TTO is scored higher than RS [7].

Whether societal preference (amongst the general population) or experience-informed preference (amongst patients) should guide policy-making remains contested [4]. Gold et al. (1996) propose that societal preferences should be used for macro-level decision-making and patient preferences for meso-level (guideline development) decision-making [13]. Utility scores derived from patients may differ from those of the general population or other specialist groups such as healthcare professionals. For example, people experiencing multiple health states give greater weight to mental health states than physical health states, compared to the scoring of the general population [4, 14]. However, some authors have raised concerns over the challenges of producing fair and balanced evaluations of health states for individuals who have personally experienced the health state or symptoms described [15].

Utility scores are widely used for priority setting and resource allocation for physical health states, but less frequently for mental health states [11, 16]. However, there is little evidence of service users’ inclusion in the development or scoring of valuations in mental health states [11]. Emphasis has been placed on the cognitive challenge that scoring health utilities poses and how some mental illnesses may limit comprehension of the task [17]. Despite these concerns, empirical studies demonstrate it is feasible to derive health utility scores from patients with severe and enduring mental illnesses such as schizophrenia [15, 17–19], bipolar disorder [20], depression [11] and affective and alcohol related disorders [15]. These studies have demonstrated that service users can discriminate by disease severity and medication side effects [20]. It has been recognised that the questions and procedures used to generate utility scores are abstract and challenging [21] and there have been recommendations that methodology should be refined to accommodate patients’ ‘mental status’ [17]. Another significant concern around framing effect biases, can be reduced through the involvement of mental health service users in developing the health state descriptions used to elicit utility scores, [7] and is central to the research approach reported in this paper.

A comparative research design, with study materials co-produced with service users, may reduce some of the limitations to help achieve more valid utility measures. Studies comparing SG and TTO are usually exclusively quantitative. Our study also includes qualitative elements to gain insight into the acceptability of the different utility methods and explore factors influencing participants’ values and preferences in health state valuations [22]. We also examine whether health utilities can be measured remotely using an e-survey.

Study objectives were:

- To co-develop descriptions of mental health states from which utility scores could be derived, and co-produce utility questions that are understandable to service users.
- To assess the feasibility and acceptability of using scenario-based health states to measure health utility.
- To determine which utility measure is preferred and how participant groups discriminate between the scenarios.
- To compare utility scores provided by service users, carers, healthcare professionals and interested members of the general public.

## Materials and methods

### Research design

The study used a cross-sectional online survey to collect quantitative and qualitative data.

### Population, sampling and data collection

Mental health service users, carers, healthcare professionals and interested members of the public were invited to take part in the survey. Service users and carers were recruited via a link on the national Rethink Mental Illness charity website (www.rethink.org). The survey was promoted by snowball emails and social networking sites Twitter and Facebook. Participants were self-selecting and indicated which participant group they identified with. The electronic survey was open for recruitment from March 2015 to July 2015. The study was conducted in the UK but did not preclude participation from other countries.

### Ethical approval

Ethical approval was granted by the School of Health Sciences’ Research Ethics Committee City, University of London.

### Instrument design

The survey was designed collaboratively with members of the Service User and Carer Group Advising on Research (SUGAR) [23] and research academics at City, University of London. The SUGAR group has 13 service users with lived experience of mental illness and three carers and meets monthly to advise on research projects within the Centre for Mental Health Research and East London NHS Foundation Trust. The original study design was presented at the January 2013 SUGAR group meeting. Members were invited to become involved in the study, to ask questions about the research and discuss how the research study could proceed. Members of the SUGAR group agreed to work collaboratively on the study and contribute to the design of instruments. Instrument design occurred in two stages: 1) developing the mental health state scenarios; and, 2) designing the survey questions.

#### Stage 1: Development of the mental health state scenarios

The SUGAR group helped write several short fictional scenarios describing the presentation and experience of specific mental health conditions. Members worked in groups of two or three. We offered guidance to the group by prompting members with questions such as ‘how would someone describe living with that condition?’ ‘What would impact on their condition?’ Once complete, each scenario was presented to the wider group for feedback. The scenarios went through several iterations through group discussions over three months. An example hypothetical health state and its description is given in Box 1.

**Box 1: Psychosis health state scenario**

*‘Joseph lives alone and is scared that people are out to kill him and says that these people are going to bomb his house. His neighbours also want him out because of what they see as strange behaviour on his part*, *his general oddity and the fact that he talks to himself. Joseph hears voices which reinforce his fears.’*

A total of ten scenarios, focusing on different mental health states, were developed. The final survey used five scenarios chosen by the SUGAR group. The example in Box 1 focusing on psychosis, and another four scenarios on medication side-effects, self-harm, eating disorders and severe depression were used (see S1 Table).

#### Stage 2: Development of the survey questions

Survey questions were designed using SG and TTO methods for scoring the five health states. The survey questions were designed by the authors and reviewed by the SUGAR group to ensure that they were comprehensible.

The survey was housed on SmartSurvey (www.smartsurvey.co.uk) and included 29 questions (some with several parts). We estimated it would take 20–25 minutes to complete. The first part asked respondents to read the information sheet and consent to the study. Participants could withdraw from the study at any stage by simply clicking out of the survey. For descriptive purposes socio-demographic information (i.e., age, gender, ethnicity, level of education and marital status) was requested and is summarised in Table 1. The main body of the survey included the five scenarios with questions linked to each of the scenarios to assess health utilities using RS, SG and TTO methods. After completing the three utility measures for all five scenarios, respondents were asked questions about the acceptability of the measures. Ten-point Likert rating scales assessed the perceived difficulty of each method. Preference for each method was reported alongside free text response boxes so that participants could expand on their responses. A final free-text response box at the end of the survey allowed for feedback on anything that may have affected their response to the questions. The Checklist for Reporting Results of Internet E-Surveys (CHERRIES) [24] was used to inform the development of the survey.

**Table 1:** Participant socio-demographic data.

	Service Users	Carers	Interested member of the Public	Healthcare Professionals	Service Users & Health Care Professionals
	(n = 46)	(n = 6)	(n = 31)	(n = 28)	(n = 5)
**Age in years**: mean (s.d.)	32 (12)	49 (17)	36 (11)	39 (12)	40 (18)
Range	17–62	19–72	18–59	21–57	29–67
**Gender**:					
Female	34 (74)	5 (83)	29 (93)	19 (68)	100 (5)
Male	12 (26)	1 (17)	2 (7)	9 (32)	
**Country**					
England	42 (91)	5 (83)	30 (97)	25 (89)	5 (100)
Wales	2 (4)				
Scotland	1 (2)	1 (17)		1 (4)	
Other country	1 (2)		1 (3)	2 (7)	
**Ethnicity**:					
English	30 (65)	4 (67)	18 (58)	17 (61)	3 (60)
Other British	7 (15)	1 (17)	4 (13)	2 (7)	1 (20)
Other White			2 (6)	1 (4)	
Asian			4 (13)	3 (14)	
Irish	2 (4)			1 (4)	
African			1 (3)	3 (11)	
Black/British	1 (2)	1 (17)			
Black/Caribbean			1 (1)		
Other ethnic group	3 (6)		1 (3)		
**Relationship Status:**					
Never married/formed a civil partnership	27 (59)		11 (35)	10 (36)	3 (60)
Married/in civil partnership	9 (20)	5 (81)	9 (29)	7 (25)	
Cohabiting	7 (15)	1 (17)	3 (10)	9 (32)	2 (40)
Divorced/Separated	2 (4)		5 (16)	2 (7)	
Widowed	1 (2)		3 (10)		
**Work Status:**					
in paid employment	25 (48)	3 (50)	17 (55)	22 (79)	3 (60)
temporarily off sick	4 (8)				
Unemployed	2 (4)	1 (17)	3 (10)		
Retired		1 (17)	2 (6)		
looking after the family, home or dependents			2 (6)		
Unable to work because of Long term disability or ill health	6 (13)				
In full time education or training	9 (20)	1 (17)	6 (19)	6 (21)	2 (40)
Other	3 (6)		1 (3)		
**Qualification:**					
Higher degree	17 (37)	2 (33)	14 (45)	17 (61)	1 (20)
Degree/degree level	10 (22)		12 (39)	10 (36)	
Other higher education below degree	4 (9)		1 (3)	1 (4)	3 (60)
A-levels/similar	9 (17)	1 (17)	2 (6)		1 (20)
GCSE/O-level/similar	5 (11)	2 (33)	2 (6)		
Trade Apprenticeships	1 (2)				
No Qualifications	1 (2)	1 (17)			

All data presented as N and (%) unless stated otherwise. Missing data for Age, N = 1, Service user and Health care professional

### Utility measures

All the utility methods generate a score from 0–1 (0: *worst possible health state*– 1: *best possible health state*). The methods of eliciting utility scores for each measure are described below:

#### Rating scale questions

For each scenario participants were asked to score the health state from 0–10 with lower values representing more negative appraisals of the health state (0: *worst imaginable health state*– 10: *the best possible health state*). In order to obtain a RS utility measure the responses given by the participant was divided by 10 to produce values between the ranges of 0–1.

#### Time trade-off questions

For each scenario respondents were asked to imagine making a choice between spending the next ten years of life in the health state described (e.g., psychosis), or 'trading' some years of life to be completely free of symptoms for the rest of their life. They were then asked to indicate the maximum number of years of their life they would be willing to trade to have complete wellness. To help with comprehension the SUGAR group suggested that some people might understand the term 'trade' as swapping, surrendering or sacrificing, this was incorporated into the description.

The choice of how many years to trade was offered incrementally, one year at a time (to a maximum of 10). A choice was required for each year to identify the point of indifference which was reached when the participant could no longer choose. The utility for the health state was calculated from the proportion of years traded at the point of indifference. For example, if someone trades 4 out of a possible 10 years of life to achieve full health, then the utility they ascribe to the health state would be 0.6 (Utility = 1 –(years traded at the point of indifference/total possible years to trade)).

#### Standard gamble (SG) questions

For each scenario respondents could choose to remain in the health state (e.g., psychosis) for the rest of their lives or take a gamble in which there was a specified risk of dying but, if they did not die, they would be fully healthy. As part of this process respondents were asked to score the maximum risk of death they would take in exchange for guaranteed full health until a point of indifference. For example, if respondents find it hard to choose whether or not they would risk a 10% chance of death for a 90% chance of full health, then their utility for that state is 0.9. If they are indifferent when there is a 90% chance of death and 10% chance of full health, then their utility for that health state is 0.1.

## Analysis

### Quantitative analysis

Utility scores were calculated in Microsoft Excel, imported into SPSS version 21 [25] and checked and cleaned by two researchers. Descriptive statistics (e.g., means + standard deviations; frequencies; percentages) were used to summarise the sample characteristics and the outcome measures (utility scores) using three different methods (RS, TTO, SG) for five health states, across three participant groups (SU, HCP, MoP). These comparisons enable an evaluation of discriminatory power. Discriminatory power is a function of three factors: the description of the health state, the utility method, and the evaluative abilities of participants. Discriminatory power is present when health states that would be expected to be scored differently are scored differently. Observing discriminatory power therefore implies that no influential biases are present (e.g., floor or ceiling effects, central tendancy bias). Conversely, a lack of discriminatory power across all participant groups raises questions about the health state descriptions and/or the utility method, whereas a lack of discriminatory power in only some participant groups suggests a lack of evaluative abilities in those groups.

#### Understanding, acceptability and preference

Understanding, acceptability and preference for the three utility scoring methods across the different participant groups was assessed using (a) the proportion of successfully completed surveys, (b) a perceived difficulty Likert scale, (c) the reported preference for the utility measures, and (d) the number of zero traders and maximal traders.

#### Statistical analysis

Inferential statistics were based on three groups (n = 105): service users (SUs; N = 46), healthcare professionals (HCPs; N = 28) and interested members of the public (MoPs; N = 31) because there were insufficient participants in other groups (Carers; N = 6, Service users and healthcare professionals; N = 5). A two-way mixedanalysis of variance (ANOVA) was conducted to determine whether there were significant differences in the way the three groups (SUs, HCPs, MoPs) scored the five health states using the three types of utility measure (RS, TTO or SG). Due to multiple testing, the level of significance (α-level) was reduced to 0.01. Tukey post-hoc tests were conducted to ascertain differences in scoring for the different scenarios, utility measures and participant groups.

One-way ANOVAs were conducted to explore differences in the perceived difficulty of each utility measure and the percentage preference scores, across groups.

#### Analysis of zero-traders and maximal traders

Zero-traders, respondents who did not trade any years of life for improved health (TTO) or gamble at any % risk of death (SG), and maximal-traders, respondents who traded the maximum amount of time (10 years) or accepted the maximum amount of risk to live in perfect health, were identified.

### Qualitative data

The free text boxes enabled participants to provide qualitative information about factors that may have influenced their responses and their preference of utility measure. A basic thematic analysis was undertaken [26] line-by-line using constant comparisons. Identified themes were independently checked by two researchers and disagreements resolved by a third reviewer.

## Results

### Sample

During the four month recruitment period 204 people accessed the survey: 85 were partially completed and 119 (58%) fully completed. The mean time to complete the survey was 14 mins (range from 4 mins to 120 mins), with 75% of respondents completing the survey within 9 and 57 minutes. Eight participants returned to the survey and time of completion could not be obtained. Participant characteristics are given in Table 1. Of the 119 complete responders, 46 identified as service users; 6 were carers; 31 were interested members of the public; and 28 were mental health professionals. Five respondents described themselves as both a service user and a healthcare professional. A further 3 participants that selected multiple identities were excluded, leaving 116 in the descriptive data. Participants were between 17 and 72 years old. More females completed the survey than males, ranging from 68%-100% across participant groups. The majority of the respondents were based in England and a large percentage (73%) identified themselves as English or “other British”. A high proportion had University-level education, with 22% reporting having a degree (e.g., BA, BSc) and 37% a higher degree (e.g., MSc, PhD).

#### Non-completion of the survey

The surveys that were started but not completed (N = 85) were not included in the analysis however we provide some further detail here. Seventy nine completed the first stage of the survey allowing us to view the socio-demographics. The majority left the survey after completing the first scenario questions. The demographics of the participants completing the survey were similar to those who did not. The average age of non-completers was 36, 45/79 were female, 44/79 had a higher degree and 24/79 were service users.

#### Comparative utility scores

Utility scores ranged from zero to one. Comparative mean scores for the utility measures and participant groups are provided in Table 2. Similar patterns of scoring were observed across participant groups and the SG consistently scored higher (indicating a better health state) than the RS in all five scenarios, and for some health states more than TTO.

**Table 2:** Comparative utility scores between utility measures used and respondents.

	Participant Groups		
Utility Measures by Scenario (Mean ± SD)	Mental Health Service User	Interested member of the public	Healthcare Professional
	(n = 46)	(n = 31)	(n = 28)
**Psychosis**			
**RS**	0.30 (0.17)	0.26 (0.22)	0.26 (0.16)
**TTO**	0.24 (0.33)	0.28 (0.29)	0.34 (0.33)
**SG**	0.50 (0.37)	0.50 (0.26)	0.52 (0.34)
**ANOVA:**			
**by Group**	F (2, 102) = 0.189, **p = 0.83**		
**by Utility**	F (1.95, 204) = 24.65, **p<0.0001**		
**Medication Side-effects**			
**RS**	0.47 (0.18)	0.53 (0.19)	0.53 (0.15)
**TTO**	0.56 (0.36)	0.65 (0.31)	0.68 (0.33)
**SG**	0.68 (0.37)	0.74 (0.33)	0.78 (0.33)
**ANOVA:**			
**by Group**	F (2, 102) = 1.67, **p = 0.19**		
**by Utility**	F (2, 204) = 22.01, **p<0.0001**		
**Self-Harm**			
**RS**	0.38 (0.20)	0.36 (0.19)	0.39 (0.20)
**TTO**	0.47 (0.39)	0.53 (0.30)	0.58 (0.31)
**SG**	0.61 (0.36)	0.67 (0.32)	0.75 (0.29)
**ANOVA:**			
**by Group**	F (2, 102) = 1.32, **p = 0.27**		
**by Utility**	F (2, 204) = 41.05, **p<0.0001**		
**Eating disorders**			
**RS**	0.35 (0.22)	0.35 (0.20)	0.41 (0.19)
**TTO**	0.42 (0.34)	0.56 (0.33)	0.63 (0.31)
**SG**	0.60 (0.34)	0.69 (0.31)	0.75 (0.28)
**ANOVA:**			
**by Group**	F (2, 102) = 3.65, **p = 0.03**		
**by Utility**	F (2, 204) = 46.85, **p<0.0001**		
**Depression**			
**RS**	0.30 (0.21)	0.36 (0.23)	0.33 (0.18)
**TTO**	0.31 (0.35)	0.48 (0.30)	0.49 (0.34)
**SG**	0.50 (0.36)	0.64 (0.29)	0.70 (0.25)
**ANOVA:** **by Group**	F (2, 102) = 4.80, **p = 0.01**		
**by Utility**	F (2, 204) = 35.67, **p<0.0001**		

There were no significant interactions between utility measure and the participant group for any scenario. There was a substantial main effect of utility measure on utility score in all scenarios, suggesting that different utility measures produce different scores. Table 2 summarises the descriptive and inferential statistics. There were no significant differences in how participant groups scored four scenarios (psychosis, side-effects, self-harm and eating disorders). There was a significant main effect of participant group in the depression scenario, F (2, 102) = 4.80, p = 0.01, partial eta squared = 0.086, suggesting that there was a difference in the way that service users, healthcare professionals and interested members of the public scored this scenario. Tukey post-hoc tests suggested that service users gave depression a lower utility score (RS: 0.30; TTO: 0.31; SG: 0.50) (perceived it as worse to live with) than healthcare professionals (RS: 0.33; TTO: 0.49; SG: 0.70), p = 0.036 and interested members of the public (RS: 0.36; TTO: 0.48; SG: 0.64), p = 0.025 (post hoc tests non-significant at reduced α level <0.01).

#### Ranking the scenarios

There was considerable consistency in how the scenarios were ranked and the type of utility measure used (see Table 3). Similarly, there was consistency across the participant groups’ mean ranking of health states.

**Table 3:** Health state scenarios ranked according to valuation by method and participant.

**Mental Health Service Users (N = 46)**			
**Rank**	**Rating Scale**	**Time Trade-Off**	**Standard Gamble**
**1**	Psychosis / Depression	Psychosis	Psychosis / Depression
**2**	Eating Disorders	Depression	Eating Disorders
**3**	Self-harm	Eating Disorders	Self-Harm
**4**	Medication side-effects	Self-Harm	Medication side-effects
**5**		Medication side-effects	
**Interested members of the public (N = 31)**			
**Rank**	**Rating Scale**	**Time Trade-Off**	**Standard Gamble**
**1**	Psychosis	Psychosis	Psychosis
**2**	Eating Disorders	Depression	Depression
**3**	Self-Harm / Depression	Self-Harm	Self-Harm
**4**	Medication side-effects	Eating Disorders	Eating Disorders
**5**		Medication side-effects	Medication side-effects
**Healthcare Professionals (N = 28)**			
**Rank**	**Rating Scale**	**Time Trade-Off**	**Standard Gamble**
**1**	Psychosis	Psychosis	Psychosis
**2**	Depression	Depression	Depression
**3**	Self-Harm	Self-Harm	Self-harm / Eating Disorders
**4**	Eating Disorders	Eating Disorders	Medication side-effects
**5**	Medication side-effects	Medication side-effects	

Rank: 1 = Worst Health State

Across all groups and utilty measures, psychosis scored as the worst health state to live with while medication side-effects were viewed most positively. MoPs and HCPs scored depression as the second worst health state across all utility measures; SUs ranked depression equal to psychosis using the RS and SG. Eating disorders and self-harm were mid-ranked across all groups and utility measures.

### Acceptability of the utility measures

#### Perceived difficulty in completing the questions

Participants were asked to measure on a Likert scale how hard they thought it was to complete the questions. A score of zero referred to ‘not difficult at all’ and a score of 10 represented ‘very difficult’. A one-way ANOVA revealed no significant differences in the perceived difficulty of the utility methods between SUs (mean = 5.35 (SD = 2.87), MoP (6.32, 2.86) and HCPs (6.75, 2.44) (F (2, 102) = 2.53, p = 0.085).

Further qualitative detail about the perceived difficulty in completing the questions was derived from the free-text responses from 38 participants (12 SUs, 1 Carer (C), 14 HCPs, 7 MoP, and 4 people identifying with two or more of the population categories). These were collated into five core themes, a summary is provided in Table 4 with illustrative quotes.

**Table 4:** Difficulty with scoring health utilities: Themes and illustrative quotes.

**Moral and emotional reactions**	
Participants referred to how they reflected on their choices and spoke about emotional reactions to the questions and moral dilemmas that they felt when completing the valuations.	
• ‘*frustrated as I couldn't explain my choices’* [C & HCP] •‘*The questions which raised isolation as a factor made me more likely to trade years*’ [HCP]	
• Another spoke about feeling ‘*despair*’ [C] • *‘I felt guilty rating things as less important as it seemed like I was belittling the condition’* [P]	
• ‘*questions difficult in a moral sense’* [HCP]	
**Relevance to own experience**	
Some participants argued that lived experience could be advantageous in answering the questions. Concerns were raised about difficulties in imagining what it would be like to live with some health state. This was acknowledged by SUs and HCPs.	
• *Could relate more to own experience so rated them worse*, *which makes my answers subjective*, *rather than objective’* [SU]	
• ‘*Difficult to understand what those symptoms really feel like and be able to accurate make a judgment as to what you would do’* [HCP]	
• *‘As only one section was even vaguely relevant to something I had experienced*, *I did not feel competent to make an assumption of what it would be like to experience most of the states described’ [SU]*	
• *‘finding it hard*, *to imagine being in the described situations’ [HCP]*	
**Standard Gamble Confusing**	
Several people found the wording in the risk question difficult	
• *‘percentage questions confusing’* [HCP]	
• *‘finding the ‘risk % section quite hard to understand’[P]*	
• *‘Risk % section quite hard to understand’* [SU]	
**Instructions unclear/ambiguous**	
Several responses were received around the wording and difficulty with interpreting what was expected when completing the valuations.	
• ‘I found the wording & the concept of the questions confusing’ [HCP]	
• Another mentioned *‘not really understanding your instructions*. *It felt very abstract*.*’ [P]*	
• ‘Instructions were complex’ [P] ‘Too complicated’ [P]	
• ‘Instructions were not clear’ [HCP]	
**Conceptually challenging/uncertainty over choices**	
Some respondents found the methods conceptually challenging and making valuations philosophically difficult. Some references were made to concerted efforts in thinking through the responses and making judgements.	
• *‘This is not a questionnaire that could easily be completed by a lay person who does not have research training’ [P]* • ‘hard to be consistent across questions’ [P]	
• *One respondent stated it was ‘hard to make a judgement between trading the end years of your life with the likelihood of dying by suicide in the next ten years’ [SU]*	
• *‘Life & death decisions are hard and not very realistic’* [SU]	

#### Utility measure preference

The majority of participants (N = 72, 60%) found the TTO measure easier to complete than the SG (N = 47, 40%). Exploring differences across participant groups revealed that preference for the TTO was held by SUs(63%, N = 29), MoP(58%, N = 18), HCPs(61%, N = 17) and 3 out of 5 (60%) people identifying as both SUs andHCPs.

There were 22 free-text responses about how the preferred choice was made. Ten were from SUs, six fromMoP, four from HCPs, and one each from a carer and a SU who was also aHCP. Nine respondents indicated that neither TTO nor SG was easier to complete, three reported that SG was easier, and four thought that the TTO was easier and gave reasons for these.

Participants who preferred the TTO measure (N = 4) provided responses that fell into two main categories:

- **Lack of clarity (of SG)**: TTO was easier to understand than the SG because *“[SG was] confusingly worded”* and *“a bit too arbitrary”*.
- **Personal meaning**: “Would rather have quality of life over duration” [service user and healthcare professional]. A service user expressed that the TTO was easier to relate to for them “*Because I know how much time in my life has been lost being ill”*.

Participants who preferred the SG (N = 3) provided responses that could be grouped into two key areas of concern.

- **Uncertainty:** not knowing the length of life years that they had left: *‘[finding] Balance between trading time for wellness is difficult to assess given none of us know how long we will live*. *Also is effected by age*. *The percentage risk is more immediate’* [SU].
- **Complexity**: A service user thought that the wording in the TTO was more difficult *“I couldn’t figure out whether I would spend 10 years unwell and then be okay for the rest of my life or 10 years and then die straight away*.*”* A mental health professional also stated that the SG was easier because they were “*short questions and easy to select the percentage’*.

#### Analysis of zero-traders and maximal-traders

Zero-traders are participants who want the maximum length of life at whatever cost to quality of life. Maximal-traders want the maximum quality of life at whatever expense to length of life. There were zero-traders and maximal-traders in both of the utility scoring methods, these will be presented in turn.

#### Time trade-off

Five participants were zero-traders across all scenarios using the TTO method (2 service user, 2 healthcare professionals and 1 member of the public). Overall there were 70 incidents of zero trading (12% of all responses) across the scenarios and participants. The highest incidence of zero traders was for the medication side-effects scenario with 24 participants (20%) choosing not to trade years. This contrasts with only 6 participants (5%) choosing not to trade years in the psychosis scenario.

Seven participants who were maximal traders across all scenarios using the TTO method (4 service users, 1 carer, 2 healthcare professionals and 1 member of the public). There were 152 incidents of maximal trading (26% of all responses) across the scenarios. The highest incidence of maximal traders was in the psychosis scenario with 58 participants (49%) choosing to trade the maximum number of years (10 years) to live in a better health state. The lowest incidence of maximal trading was for medication side effects with 16 people (13%) choosing to trade 10 years to live without side effects.

#### Standard gamble

Three participants were zero-traders across all scenarios using the SG method and all were service users. Overall there were 69 incidents of zero trading (12% of all responses) across the scenarios and participants. The highest incidence of zero traders was for the medication side-effects scenario with 26 participants (22%) choosing not to accept any % risk of death for a better health state. This contrasts with only 6 participants (5%) choosing not to risk death in the psychosis scenario.

Six participants were maximal traders across the scenarios using the SG method (3 service users, 1 carer and 2 members of the public). Overall there were 62 incidents of maximal trading (10%) across the scenarios. The psychosis scenario had the highest incidence of maximal traders (17/119 (14%)), while medication side effects had the lowest incidence of maximal trading (11/119 (9%)).

#### Did anything else affect participants’ responses?

We received 66 participant responses to the open-ended question asking if anything had affected their responses. The majority of the responses (N = 46, 70%) related to personal experiences of mental illness and identifying with the person in the scenario. Twenty four of the responses about personal experience were from service users, with seven from HCPs, six fromMoP, three from carers, and five responses from people identifying with two or more of the participant type categories.

*“Probably those ones that I can 'feel' the pain of relative to those ones that I have to imagine—I'm probably more willing to trade years on things that I can remember feeling*.*”* [SU]

*“Partner suffers from psychosis and I have seen this suffering straight on”* [C]

*“I have experienced severe depression myself & have also worked with people with the rest of the diagnoses discussed*.*”* [HCP]

In contrast, others reflected on their lack of personal lived experience of mental illness and how that bought challenges in completing valuations on the health states.

*“Not having first-hand experience and therefore having to rely on impressions of my personality to consider what my actions might be”* [MoP]

Several respondents referred to their emotional state at that moment (N = 7), mentioning feelings of sadness, tiredness and social isolation. Whilst two people reflected on the complexity of the scenarios and others on challenges with moral decisions and two service user participants referred to negative images or stigma of mental health conditions.

## Discussion

In this study, we sought to collaborate with service users to co-produce descriptions of mental health states from which to generate utility scores and frame utility questions so that they are comprehensible to service users. Another aim was to determine the feasibility of using different utility methods via an online questionnaire. We compared utility scores provided by service users, healthcare professionals, members of the public, and carers (descriptively). The acceptability of the co-produced health states and the different utility methods to determine health utility was also examined.

The results indicated that:

1. Mental health state scenarios used to generate utility scores can be co-produced and are well received by a broad range of participants using an online survey.
2. Standard techniques used to elicit utility valuations (SG; TTO and RS) were feasible for use with service users, carers, healthcare professionals and members of the general public.
3. Similar trends were seen in utility scores elicited by the different utility methods across all participant groups. For a given health sceanrio, the SG was generally scored higher (indicated a more preferred health state) compared the TTO and RS. Some differences between participant groups emerged in the scenario on depression.
4. Participants ranked the scenarios comparably demonstrating equivalence in discrimination and weighting of the scenarios.
5. The TTO was preferred over the SG.

Searching the literature we were unable to locate previous examples where mental health state scenarios were co-produced with service users and carers for use within an e-survey.

In line with previous research [8], we found significant differences between the utility scores when using different types of utility measure within each scenario. Similar patterns to those found in other studies were identified, with respondents scoring the highest utility when using the SG and lowest utility when using the RS methodology [27].

Of particular interest, service users gave a lower utility value (indicating a less preferred health state) for the depression scenario than healthcare professionals and interested members of the public. Isacson et al. (2005) found that people with depression rated their health state utilities significantly lower than those without [28]. The literature to date suggests that “well-informed” respondents (i.e., people who have experienced the condition) may score the scenario as less threatening and therefore give a higher utility score than respondents who did not share that experience. This is the converse of what is seen in this data, and therefore does not fit with theories such as the disability paradox [29] or the stress-appraisal-coping paradigm [30]. It is important to acknowledge that we do not know which of our respondents had experienced depression and therefore it is unclear whether these findings are due to direct experiences or knowledge relating to a hypothetical health state. Stiggelbout (2008) provides a thorough review on how scenarios are interpreted and judgements are made by people with lived experience and those naïve to the lived experience during the process of scoring utilities [4]. Of particular interest to the field of mental health is the focus of the illness in the person’s life and their constructed meaning. One study showed a recovery-focused approach to interpreting the illness where people with the human immodeficiency virus (HIV) reframed living with the illness positively by focusing on how HIV fit in with the broader context of their life rather than purely focusing on the impact on their health [31].

### Ranking

With regard to how the scenarios were ranked, there was consistency across participant groups in ranking the psychosis scenario as the most undesirable scenario to live with. This was irrespective of utility measure used and it may have implications for service users prioritising treatments that could maximize preferences or health gain. Of course, prioritisation will depend on the estimated gain from any actual intervention.

### Acceptability

#### Completion of the survey

In this pilot study a high proportion of service users, healthcare professionals and members of the public successfully completed the utility scores for five described healthcare scenarios. There were no substantial differences demographically between participants who completed the survey and those that did not.

#### Difficulty

In terms of difficulty in completing the online survey, we found that there was a suggestion that healthcare professionals and members of the general public perceive the utility measures as more difficult than service users. However there were no significant between-group differences. Arguably this demonstrates that mental health service users are just as capable of scoring utility scenarios as are members of the public and healthcare professionals. However, this interpretation should be treated cautiously as the qualitative data suggests that there is some difficulties with the SG and TTO utility methods for all participant groups. Participants found the scenarios and scoring mechanisms difficult to understand and were uncertain over how to score the scenarios overall. Some respondents also had concerns around accepting the philosophical notions of trading, ‘giving up life years’ or ‘risking’, indicating that face validity within the scenarios remains a challenge. Consistent across the groups was a preference for scoring the TTO. Participants found the TTO easier to understand as they were able to relate to losing years of life more readily than accepting an increased risk of death (SG).The literature also suggests that the TTO is preferred by some for the relative ease of use compared to the SG and has been reported as consistent with individual preferences [9, 12] and the most frequently used method [14].

#### Zero-traders

Only 5% of participants using both TTO and the SG for psychosis refused to trade. Zero-traders were most prevalent in the scenario for medication side effects with 20% refusing to trade time (TTO) and 22% unwilling to gamble on an increased risk of death (SG). This may be a function of participants accepting the side effects as a necessary albeit an undesirable aspect of treatment.

#### Limitations

Some participants started the survey but did not complete it (non-completers). The reason for this is unknown, although feedback from other respondents suggest it may have been due to the format of the survey and complexity of questions.

Recruitment was voluntary using an internet link, some degree of self-selection bias is likely and probably resulted in a less representative sample. Table 1 indicates that the sample of service users is unusually well-educated, with 37% having a higher degree and another 22% a degree. This is higher than the average in the UK, where 34.4% of the population is estimated to have achieved a degree-level qualification or above [32]. Given the nature of the research it may not be surprising that the sample is relatively well educated, and does limit the generalizability of the findings. The use of online surveys can also pose a challenge for people who do not have access to a computer and this may have had an impact on recruitment. However it is difficult to estimate the true impact of any potential selection bias when data on non-participants is unavailable [33].

Respondents identified themselves as being healthcare professionals, members of the public, service users or carers, responses which cannot be verified by the researchers. Additionally for many participants these categories are not exclusive and there will be overlap with people identifying with more than one category. For those who identified as service users we have no information about their clinical condition (e.g. diagnosis, severity, duration of time living with the condition) and therefore associations with scoring disease specific-scenarios was not possible [17]. Because we did not use quality of life measures alongside the utility measures convergent validity could not be assessed.

In this study we did not control for the order effects of scenario presentation and the potential that scores were moderated by anchoring.

There are also unresolved questions about how to measure health. Our measures informed by our co-produced scenarios also included an element of social participation. This is an important consideration when proposing to measure mental health with people whose condition, recovery [34] and quality of life is affected by broader social considerations such as housing or employment experiences and interventions. One of our scenarios included the description of neighbours’ perceptions of the person with the illness, which may reflect real issues around relating to inclusiveness, stigma and even the reality of experiencing paranoia, but here there is also a danger of stretching the concept of social participation.

It may be argued that these types of analyses lend themselves more to moderate disorders, the treatment for which is typically designed to ameliorate symtoms as part of improving mental health. With a recovery model in prominence [34], symptom control may not always be the sole concern for severe and enduring disorders such as schizophrenia, where many interventions would seek to target quality of life much more broadly (including housing, employment and other measures of recovery and social participation). Biases in the direction of understating the benefits of these factors on the quality of life of individuals could arise and this may be a further limitation.

In addition, some conditions are not susceptible to adaptation, and they interrupt daily life almost continually. By their very nature they draw attention to themselves (one cannot just think about something else most of the time); for example, the pre-occupative nature of depression or chronic pain. With this in mind, service users may give depression a lower utility score (i.e. less preferred health state) than other groups, with a risk thereafter for utility weights to be given that are too high. Additionally every preference elicitation question, by their nature, focuses our attention on something, and so we will generally be led to overstate the relative importance to our lives of the things that we are asked to focus on [35]. For equal consideration is the evidence that suggests that the strength of preference may also be a poor guide to the intensity of experience [36–38] and a propensity for us to exaggerate the extent to which we will attend to the state being valued (Dolan and Kahneman, 2008), with us all being members of the ‘public’ and ‘patients’ and therefore susceptible to exaggeration [35].

Dolan et al. (2010) also point out, trade-off responses themselves are related to the frequency and intensity of negative thoughts about health in ways that may not have been previously well captured by any of the proposed valuation methods [35]. Different values may also capture “experience” rather than “preference.” Dolan and Kahneman (2008) cite Smith et al’s (2006) work with the following example; a patient with a colostomy thinks they are happy with the colostomy, and expects to be happy again without it. However, when it is removed they remember their previous state (of having the colostomy) as being unacceptable and, in terms of preferences, they report that they would be willing to pay a great deal, including life-years, to get rid of that state [39], a reflection of the extremely negative prior experience.

Lastly it is worth considering in the treatment of mental health preferences the potential for ‘cognitive denial’, where patients may find it difficult to admit how poor their health really is, or ‘suppressed recognition of full health’ where patients cease to realize what full health may be like and a have ‘lowered expectation’ overall. [40].

#### Future development of utility measures

Future research of this type may provide a more rigourous assessment of how health is being conceptualized in the development of such scenarios, while finding ways of helping to create scenarios and scoring mechanisms that are less complex. Possibly a greater challenge will be to create scenarios that do not lead to philosophical objections.

Nord et al. [41] discuss the use of QALYs in terms of ex ante and ex post. Ex ante is the more traditional approach and refers to health utility judgements made by the general public from behind a ‘veil of ignorance’. There is merit in the ex post approach which refers to the utilization of direct experience of the health state as “experienced utility”. The participants within this study are a combination of both ex ante and ex post participants. It may be beneficial to identify previous health experiences in respondents but conversely it may influence willingness to participate if scenarios are felt less ‘hypothetical’.

## Conclusion

This study involved service users and reports the initial steps towards developing and embracing a process of research co-production in a complex field [42, 43]. Additional studies involving service users in utility measurement are needed in the attempt to promote sensitive measurement design, increase instrument validity, study feasibility and the acceptability of the measures. Future studies may aim to build on more extensive involvement by developing knowledge and understanding to include service users in the analysis of data and interpretation of results [44].

Traditionally there have been wide variations in the utility values reported contributing to an overall lack of clarity in reporting methods used to elicit the utility values [45]. This study offers data to compare different valuation methods in order to help assess their feasibility whilst at the same time transparently reporting the methods and some of the difficulties and limitations of our approach. It adds to the limited qualitative evidence reported alongside utility scores for a range of health states and offers insights into factors that influenced respondents’ decisions, the relative difficulty of and preferences for measures used. This will help inform our future research and that of others to better prepare such utility design in the future.

## Supporting information

S1 Table Available as information file for the five scenarios used in the utility measurements and offered to participants in the online questionnaire. (DOCX)
