# Supplementary material for: What utility scores do mental health service users, healthcare professionals and members of the general public attribute to different health states? A co-produced mixed methods online survey
Source: PLoS One. 2018 Oct 23;13(10):e0205223. doi: 10.1371/journal.pone.0205223 (PMC6198969; doi:10.1371/journal.pone.0205223)
Supplement: S1 Table — (DOCX) [file pone.0205223.s001.docx]

|  |
| --- |
| **Supporting Information (S1 Table)** |
|  |
| **The five scenarios used in the utility measurements and offered to participants in the online questionnaire** |
| 1) Psychosis/paranoia health state scenario |
| Joseph lives alone and is scared that people are out to kill him and says that these people are going to bomb his house. His neighbours also want him out because of what they see as strange behaviour on his part, his general oddity and the fact that he talks to himself. Joseph hears voices which reinforce his fears. |
| 2) Side effects of medication health state scenario |
| Manji has a diagnosis of schizophrenia and has been compliant with his intra-muscular depot injection treatment. This injection is an anti-psychotic medication and has caused Manji to put on a lot of weight recently. This has affected his general psychological well-being as he does not feel good about now being 2 stone heavier that he was a year ago. |
| 3) Self-harm. Poor body image |
| George grew up in a large family and was bullied by his siblings and by other children at his school. He was called a weakling by them because he was thin and weak. As he became older he found he avoided his reflection and that he hated his old body image. He expressed hating his body and feeling ashamed of it. He felt it was normal to cut himself and he felt this was normal and a way of releasing his uncomfortable emotions. His forearms were marked by scars where he had self-harmed. |
| 4) Eating Disorder |
| Lily is 16 years old and studying hard for her A levels which she is finding very stressful. She says she feels worthless and alone. She is 7 stone and probably two stone under her ideal weight. However she expresses feeling fat and plain looking and finds it hard to eat at all some days. Some days Lily has dizzy spells and increasingly she will leave a lot of her meal or skip it all together. Her mother is concerned that she may be losing more weight and has discovered packets of empty laxatives, which she believes Lily is using to help lose further weight. |
| 5) Depression |
| Johnny feels worthless. He can’t eat and sleeps badly. He wakes very early but finds it impossible to get up and get going. His past haunts him and he has feelings of guilt and shame. No one contacts him and he contacts no-one. Simple tasks like shopping he finds very difficult and he feels like he gives off an aura of darkness. Tears come easily for no apparent reason. Some friends have urged him to pull himself together and have suggested he enroll for evening classes. These friends worry that he has changed from an out-going person to one who is completely withdrawn. |
